# Inter-related mechanisms of engagement in adaptive yoga for acquired brain injury: a qualitative implementation mapping study

**DOI:** 10.3389/frhs.2026.1879725

**Published:** 2026-08-06

**Authors:** Jennifer A. Weaver, Jessica Bombino-Elliott, Janell Pisegna, Alec M. Grove, Jaclyn A. Stephens, Arlene A. Schmid

**Affiliations:** 1 Department of Occupational Therapy, College of Health and Human Science, Colorado State University, Fort Collins, CO, United States; 2 Department of Occupational Therapy, College of Health Professions, Virginia Commonwealth University, Richmond, VA, United States; 3 Department of Health and Exercise Science, College of Health and Human Science, Colorado State University, Fort Collins, CO, United States

**Keywords:** brain injury, implementation science, qualitative, theoretical domains framework, yoga

## Abstract

**Background:**

Acquired brain injury (ABI) is associated with chronic physical, cognitive, emotional, and social impairments requiring long-term rehabilitation. Adaptive yoga is an evidence-based intervention that may address these needs; however, implementation remains limited. Thus, determinants of engagement must be translated into actionable, theory-informed implementation strategies. This study aimed to (1) identify determinants of engagement in adaptive yoga for individuals with ABI using the Theoretical Domains Framework (TDF), and (2) operationalize these determinants into mechanisms and implementation strategies.

**Methods:**

We conducted an implementation mapping study using qualitative data from participants (*n* = 9) who completed an 8-week adaptive yoga randomized controlled trial. Data were collected through focus groups and semi-structured interviews and analyzed using the framework method. Inductive *in-vivo* coding was followed by deductive mapping to TDF domains. Determinants were aligned to the Capability, Opportunity, Motivation–Behavior model to specify mechanisms of action and mapped to implementation strategies using the Expert Recommendations for Implementing Change (ERIC) taxonomy. Strategies were further specified by actor, action, target, temporality, and dose.

**Results:**

Qualitative data mapped to 12 TDF domains. Knowledge and Environmental Context and Resources represented barriers related to communication, scheduling, and navigation. Skills, Social Influences, Emotion, and Beliefs about Capabilities facilitated engagement. Determinants influenced engagement through three inter-related mechanisms: (1) adequately trained personnel, (2) adaptable yet structured program delivery, and (3) socially driven group processes. These mechanisms reflect capability, opportunity, and motivation-related processes through which 12 ERIC implementation strategies are expected to act.

**Conclusions:**

Engagement in adaptive yoga for individuals with ABI is shaped by inter-related mechanisms rather than isolated determinants. Linking determinants, mechanisms, and strategies provides a theory-informed pathway for intervention refinement and future hybrid effectiveness-implementation testing.

## Introduction

Traumatic and non-traumatic brain injuries [i.e., acquired brain injuries (ABI)] are associated with chronic physical, cognitive, emotional, and social impairments that often require long-term rehabilitation (1–7). Although adaptive yoga has facilitated improvements in participants' balance, mobility, self-efficacy, and quality of life following ABI, access to these programs remains limited (8–12). Individuals with ABI encounter numerous barriers to participating in community-based rehabilitation, including depression, fatigue, concerns about safety, physical limitations, reduced confidence to exercise, transportation challenges, and inaccessible environments (13, 14). Understanding the determinants (i.e., barriers and facilitators) that influence engagement in adaptive yoga is an important step towards improving participation and informing future implementation efforts.

Adaptive yoga has demonstrated promising clinical outcomes across several pilot and feasibility studies (8–12, 15). However, little research has examined the determinants of participant engagement needed to inform the next stage of intervention testing (16–18). As rehabilitation research increasingly emphasizes progression from feasibility studies to hybrid effectiveness-implementation trials, understanding the determinants that influence participant engagement has become essential for selecting and tailoring implementation strategies (19, 20).

Theory-informed approaches are needed to move beyond descriptive lists of barriers toward actionable implementation strategies. The Theoretical Domains Framework (TDF) identifies behavioral determinants influencing implementation (21, 22), the Capability, Opportunity, Motivation–Behavior (COM-B) model specifies mechanisms through which these determinants influence behavior (22–24), and the Expert Recommendations for Implementing Change (ERIC) taxonomy provides implementation strategies that target these mechanisms (19). Together, these complementary frameworks offer a systematic approach to translating qualitative findings into testable implementation strategies for future hybrid effectiveness-implementation trials (19). Accordingly, the primary aim of this study was to use the TDF to identify determinants influencing engagement in adaptive yoga among adults with ABI. A secondary aim was to link these determinants to COM-B mechanisms and ERIC implementation strategies (20, 25) to inform a future hybrid effectiveness-implementation trial.

## Methods

### Study design

We conducted an implementation mapping study (20, 25, 26) using the framework method (21, 27) to examine qualitative data collected during the final two weeks of an 8-week pilot randomized controlled trial (RCT) (28, 29). The target behavior of interest was participant engagement in adaptive yoga. We examined engagement qualitatively and considered how participant-reported determinants may inform future implementation outcomes, including acceptability, adoption, appropriateness, feasibility, fidelity, and sustainability (21). Because we aimed to identify implementation strategies, we followed the recommendations for specifying and reporting implementation strategies (20) and the Consolidated criteria for reporting qualitative research (30).

### Recruitment & enrollment of participants

Participants were drawn from the adaptive yoga arm of the parent RCT (28, 29). Eligible RCT participants were adults with chronic ABI ≥6 months and self-reported balance problems of moderate or greater severity. We used criterion-based sampling (31, 32) and invited the 12 participants who completed the yoga intervention to participate in interviews or focus groups during the final two weeks of the trial. To encourage participation, dinner was provided. Given the focused study aim, use of an established theoretical framework, and relatively homogeneous sample of participants who completed the intervention, the sample size was considered sufficient (33).

### Intervention

The adaptive yoga intervention was delivered in person for 1 h, twice weekly, for 8 weeks (28, 29). The intervention was developed for adults with ABI and included physical postures, breathwork, and meditation. Sessions were led by an experienced adaptive yoga instructor and supported by trained intervention assistants who provided environmental setup, safety monitoring, physical support, and posture modifications.

### Data collection & management

Demographic information including age, sex, time since injury, and mechanism of injury were collected by a trained research assistant and reviewed for accuracy by the study PI (JAS) at time of enrollment into the RCT.

Interview guides were developed to explore their experiences, barriers, and facilitators that influenced their engagement in the adaptive yoga intervention. The semi-structured interview guide was designed to elicit determinants influencing engagement and was informed by implementation science and behavioral theory (21, 34). Interview questions were structured to capture influences consistent with TDF domains, while allowing flexibility for participants to introduce unanticipated factors (21).

We completed focus groups and to maximize participation, we also conducted individual interviews based on participant preference and availability. Interviews were conducted either virtually or in-person in a private room by the first author (JAW) or study personnel trained by the first author. Focus groups and interviews were audio-recorded, transcribed, de-identified using pseudonyms, and checked for accuracy. We documented transcript changes in an audit log.

#### Trustworthiness strategies

Trustworthiness was supported through transcript review, memo-writing, team-based coding, consensus meetings, reflexive discussions, and documentation of analytic decisions in an audit trail (35). The interdisciplinary team included occupational (JAW, JP, JAS, AAS) and physical therapists (JABE, AMG), implementation science experts (JAW, JP), an intervention researcher (AAS), and qualitative research experts (JAW, JP), all of whom have prior experience working with individuals with ABI.

### Data analysis

We used descriptive analysis for all demographic data. Qualitative data were analyzed using the framework method, which supports both inductive and deductive analysis (27). Qualitative analyses were completed using NVivo 14 software (Version 14, https://lumivero.com/products/nvivo/). The first author led the analysis with two primary analysts, who met weekly to review codes, refine the codebook, and resolve coding discrepancies through consensus (36). Transcripts were independently coded until agreement exceeded 80% (32). Coding decisions, code definitions, and TDF domain alignment were documented in an analytic audit trail (35). Representative quotes are presented in the tables.

#### Identifying barriers and facilitators using the theoretical domains framework

Data were first coded using inductive *in-vivo* coding (36) and then deductively mapped to the 14 TDF domains (21, 22). New categories were generated when participant statements did not align with a TDF domain. We then examined whether each determinant functioned primarily as a barrier or facilitator to participant engagement in yoga (21, 34). TDF domain importance was determined based on the frequency, richness of coded data, strength of expressed perceptions, and relevance to the development of actionable implementation strategies (21).

#### Specifying implementation strategies to support implementation outcomes

Prioritized TDF domains were mapped to COM-B components to identify capability, opportunity, and motivation-related mechanisms influencing engagement. We then selected ERIC implementation strategies most likely to address identified barriers and strengthen facilitators (19, 20, 25, 37). Strategies were conceptually clustered (38) and specified by actor, action, target, temporality, dose, and candidate outcome (20) affected to support future testing in a hybrid effectiveness-implementation study.

## Results

### Participants

Nine of the 12 adaptive yoga participants completed interviews. Participants ranged in age from 29 to 82 years, with time since injury ranging from 6 months to 59 years. We completed two focus groups and two interviews. Participant characteristics are summarized in Table 1.

**Table 1:** Yoga participant characteristics.

Characteristics	*n* (%) or mean (SD)
Sample Size, *n*	9
Age, mean(SD), years	61.6 (15.5)
Time Since Injury, mean (SD), years	16.4 (19.3)
Sex, *n* (%)	
Female	6 (67)
Male	3 (33)
Education Level, *n* (%)	
Associates	1 (11)
Bachelor's	4 (45)
High School	0 (0)
Master's	1 (11)
PhD	2 (22)
Vocation	1 (11)
Etiology, *n* (%)	
Non-Traumatic Brain Injury	5 (56)
TBI	4 (44)
Race, *n* (%)	
White	9 (100)

### Identifying barriers and facilitators using the theoretical domains framework

Qualitative statements mapped to 9 TDF domains and one additional category, “Recommendations.” Knowledge and Environmental Context and Resources were the most prominent barriers, reflecting challenges with communication, scheduling, and navigation (Table 2). Skills, Social Influences, Emotion, Beliefs about Capabilities primarily functioned as facilitators, reflecting participants' perceived ability, social support, enjoyment, and confidence. These domains mapped across COM-B components, with capability shaped by Skills and Beliefs about Capabilities, opportunity shaped by Knowledge and Environmental Context and Resources, and motivation shaped by Social Influences and Emotion.

**Table 2:** Key barriers and facilitators mapped to ERIC implementation strategies.

Barrier/facilitator exemplary quote	Theoretical domain framework as a qualitative category defined by Cane et al. (2012) (22)	ERIC implementation strategy & definition (Powell et al., 2015) (19)
Key barriers and facilitators that mapped to implementation strategies clustered around “Train and Educate stakeholders”		
Barrier: “It would be easier if there was just one person helping me the whole time, the same person texting me. They changed by several people, and that was really hard. It was like who am I talking to? And why? Where am I going? What time? Wait, you told me I didn't have to fast but that person told me I had to fast but I didn't fast. I mean, it was very confusing” (Sheryl)	**Environmental Context and Resources:** Any circumstance of a person's situation or environment that discourages or encourages the development of skills and abilities, independence, social competence and adaptive behavior.	**Develop Education Training Materials:** “Develop and format manuals, toolkits, and other supporting materials in ways that make it easier for stakeholders to learn about the innovation and for clinicians to learn how to deliver the clinical innovation.”
Barrier: “If it's not written, it doesn't exist” (Kevin)	*Recommendation: Comments and recommendations for changes in intervention/program	
Facilitator: “I had things where I was extremely challenged, but it was allowed that I couldn't do this, and then there were other things, what I could do well.” (Denise)	**Knowledge:** An awareness of the existence of something	**Make Training Dynamic:** “Vary the information delivery methods to cater to different learning styles and work contexts, and shape the training in the innovation to be interactive”
Barrier: “My challenge was I couldn't remember all of the assistants' names…that was a huge stress for me…It would have been helpful to have name tags” (Anna)	**Environmental Context and Resources:** Any circumstance of a person's situation or environment that discourages or encourages the development of skills and abilities, independence, social competence and adaptive behavior	**Distribute Educational Materials:** “Distribute educational materials (including guidelines, manuals, and toolkits) in person, by mail, and/or electronically”
Barrier: “Well, and I have tons to say about this. Because I had some bad experience with directions with time with fasting without fasting. And that was incredibly frustrating for me. It's hard enough for me. But when that kind of stuff when that kind of thing happens. It's brutal for me, and it made me very frustrated. And even my last test of my heart rate. I received an incorrect address. And I ended up somewhere else. And I didn't even report that to anybody because I was so exhausted by the whole process… I know, they get a handout sheet of dates and times, maybe, you know, a map of the building and where you go, or turn, where you should park?” (Sheryl)	*Recommendation: Comments and recommendations for changes in intervention/program	
Barrier: “He [the MRI tech] made…teenager jokes. I remember how difficult it was for me to deal with that. It took my safety away because after the stroke, I am not so confident. I needed the safety that somebody says, “Don't worry about this. We take care of you.” (Denise)	**Emotion:** A complex reaction pattern, involving experiential, behavioural, and physiological elements, by which the individual attempts to deal with a personally significant matter or event	**Use train-the-trainer strategies:** “Train designated clinicians or organizations to train others in the clinical innovation”
Facilitator: “Yeah… honoring my body… we started out and move through it, it built… I really pushed myself… and it was really good.” (Anna)	**Skills:** An ability or proficiency acquired through practice	
Facilitator: “When I couldn't get up, they would help you.” (Jim)	**Skills:** An ability or proficiency acquired through practice	
Facilitator: “I liked the positive attitude that's throughout the session. I've been pleased with that” (Kevin)	**Skills:** An ability or proficiency acquired through practice	
Facilitator: “there was no judgment, no guilt, no disqualification…no critical comments or anything…I really appreciated that…I think that was the best part of adaptive yoga” (Anna)	**Social influences:** Those interpersonal processes that can cause individuals to change their thoughts, feelings, or behaviors	
Facilitator: “I thought, ‘Oh. I can do it. No problem at all.’” (Denise)	**Optimism:** The confidence that things will happen for the best or that desired goals will be attained	**Conduct ongoing training:** Plan for and conduct training in the clinical innovation in an ongoing way
Facilitator: “I prefer to be in a group… there were like, eight of us… and that's perfect.” (Patricia)	**Social influences:** Those interpersonal processes that can cause individuals to change their thoughts, feelings, or behaviors	
Barrier: “I didn't realize that it was adaptive yoga until we started.” (Patricia)	**Knowledge:** An awareness of the existence of something	**Conduct educational meetings:** “Hold meetings targeted toward different stakeholder groups (e.g., providers, administrators, other organizational stakeholders, and community, patient/consumer, and family stakeholders) to teach them about the clinical innovation”
Key barriers and facilitators that mapped to implementation strategies clustered around “Evaluate and Use Iterative Strategies”		
Facilitator: “The challenge with having a brain injury is finding accessible, affordable resources.” (Anna)	**Social influences:** Those interpersonal processes that can cause individuals to change their thoughts, feelings, or behaviors	**Conduct local needs assessment:** “Collect and analyze data related to the need for the innovation”
Facilitator: “ …the consistency, like you having it on specific days at specific times. I liked that. That helped me. Like keep a routine. It was super helpful” (Miranda)	**Knowledge:** An awareness of the existence of something	**Obtain and use patient/consumer feedback:** “Develop strategies to increase patient/consumer and family feedback on the implementation effort”
Facilitator: “Yoga is movement and movement with intention…the whole thing is that it has moved me into making the movements and everything habit, which is good.” (Anna)	**Intentions:** A conscious decision to perform a behavior or a resolve to act in a certain way	
Barrier: “Like what Jackie does, how she texts us every morning. Maybe they could do that. Because I'm with you, I was confused [re: pre-assessment directions]. I luckily, was trying to stay consistent, and I'm just checking my email. But I kept getting confused on the day that I was supposed to fast and not fast…I really need text message reminders” (Miranda)	**Knowledge:** An awareness of the existence of something	
Barrier: “I was confused by that instruction initially. I asked [in the second session], ‘Can we get handouts so we could practice in between sessions?’ She said, ‘No, I prefer we just do it in class.’ About two sessions later, she started talking about what have you been practicing that you enjoy.” (Kevin)	**Environmental Context and Resources:** Any circumstance of a person's situation or environment that discourages or encourages the development of skills and abilities, independence, social competence and adaptive behavior.	**Develop and organize quality monitoring systems:** “Develop and organize systems and procedures that monitor clinical processes and/or outcomes for the purpose of quality assurance and improvement”
Key barriers and facilitators that mapped to implementation strategies clustered around “Adapt and Tailor Content”		
Barrier: “And if I have to make a commitment for 10 weeks-it's easy to sign up for the first 8” (Denise)	**Knowledge:** An awareness of the existence of something	**Promote Adaptability:** “Identify the ways a clinical innovation can be tailored to meet local needs and clarify which elements of the innovation must be maintained to preserve fidelity”
Barrier: “I think it would be a good system if you would say we have groups from six weeks and then there's two weeks off, and then there's another group.” (Jim)	**Knowledge:** An awareness of the existence of something	
Facilitator: “It's really awesome. To observe the changes and development in my life. It was wonderful to see.” (Anna)	**Knowledge:** An awareness of the existence of something	
Facilitator: “It was a safe place here. I thought before maybe I start with yoga, this will help me to improve, but I didn't feel safe and I didn't really want to go to my gym and then to say, ‘Oh, I had a stroke or something.’ I was really looking forward to coming here because I felt safe. You couldn't fail. You could just improve.” (Denise)	**Beliefs About Consequences:** Acceptance of the truth, reality, or validity about outcomes of a behavior in a given situation	
Facilitator: “I was very surprised that I…I said, ‘Oh, you can do this?’…I could allow myself not to be perfect.” (Denise)	**Beliefs about Capabilities:** Acceptance of the truth, reality, or validity about an ability, talent, or facility that a person can put to constructive use	
Facilitator: “The energy from participants really makes a difference. Everyone in there is brain injured, and we're all in there to get some type of help with that.” (Barbara)	**Emotion:** A complex reaction pattern, involving experiential, behavioural, and physiological elements, by which the individual attempts to deal with a personally significant matter or event	
Facilitator: “I felt really safe and I could be a fragile person… that had so big impact on my emotions.” (Denise)	**Emotion:** A complex reaction pattern, involving experiential, behavioural, and physiological elements, by which the individual attempts to deal with a personally significant matter or event	
Facilitator: “I've really benefited a lot from this…let's take it slow. Let's do it on your pace. I liked that.” (Patricia)	**Skills:** An ability or proficiency acquired through practice	
Facilitator: “There was some familiarity and consistency with the people that were here and how it was structured.” (Anna)	**Environmental Context & Resources:** Any circumstance of a person's situation or environment that discourages or encourages the development of skills and abilities, independence, social competence and adaptive behavior.	
Key barriers and facilitators that mapped to implementation strategies clustered around “Engage Consumers”		
Barrier: “I need more consistency, a longer time period. I was starting to get some strength back, and that felt really good.” (Barbara)	*Recommendation: Comments and recommendations for changes in intervention/program	**Intervene with patients/consumers to enhance uptake and adherence:** “Develop strategies with patients to encourage and problem solve around adherence”
Barrier: “I really need text message reminders” (Miranda)	*Recommendation: Comments and recommendations for changes in intervention/program	
Facilitator: “I'm going to add some yoga routines… and work with my wife… and we'll work out some things.” (Kevin)	**Intentions:** A conscious decision to perform a behavior or a resolve to act in a certain way	
Facilitator: “I like the consistency of the routine” (Anna)	**Knowledge:** An awareness of the existence of something	**Obtain and use patients/consumers and family feedback:** “Develop strategies to increase patient/consumer and family feedback on the implementation effort”
Key barriers and facilitators that mapped to implementation strategies clustered around “Change Infrastructure”		
Barrier: “I had the same problem. My wife and I… GPS… we ended up where they were storing horses and goats… realizing that we were in the backside of it, and not the front.” (Kevin)	**Environmental Context & Resources:** Any circumstance of a person's situation or environment that discourages or encourages the development of skills and abilities, independence, social competence and adaptive behavior.	**Change Record Systems:** Change records systems to allow better assessment of implementation or clinical outcomes
Facilitator: “but I liked about it is the… consistency like you having it on specific days at specific times. Yep. I liked that. Okay, that helped me. Like keep a routine. It was super helpful.” (Miranda)		
Barrier: “The [MRI] room was horrible. I mean, there were stacks of stuff. And I was like, there's a big door there. Like it's a refrigerator. Oh my god. Get me outta here. The sheet was dirty. Oh, it was horrible.” (Sheryl)	*Recommendation: Comments and recommendations for changes in intervention/program	
Barrier: “The have room with a body scan. [It] was like they threw it in a closet or something. It didn't seem professional at all and I thought what am I getting into? For seven years I've been in hospital rooms, and they were clean. I just I'd lost my confidence… it just it did not seem professional” (Kevin)	**Emotion:** A complex reaction pattern, involving experiential, behavioral, and physiological elements, by which the individual attempts to deal with a personally significant matter or event	

### Specifying implementation strategies

We mapped the facilitators and barriers to 12 of the 73 distinct ERIC implementation strategies. We organized the 12 strategies into 5 of the 9 pre-existing clusters (38). The five clusters that our data mapped to were: “Train and Educate Stakeholders,” “Evaluate and Use Iterative Strategies,” “Adapt and Tailor Content,” “Engage Consumers,” and “Change Infrastructure” (Table 2).

### Inter-related mechanisms

**Figure 1:**
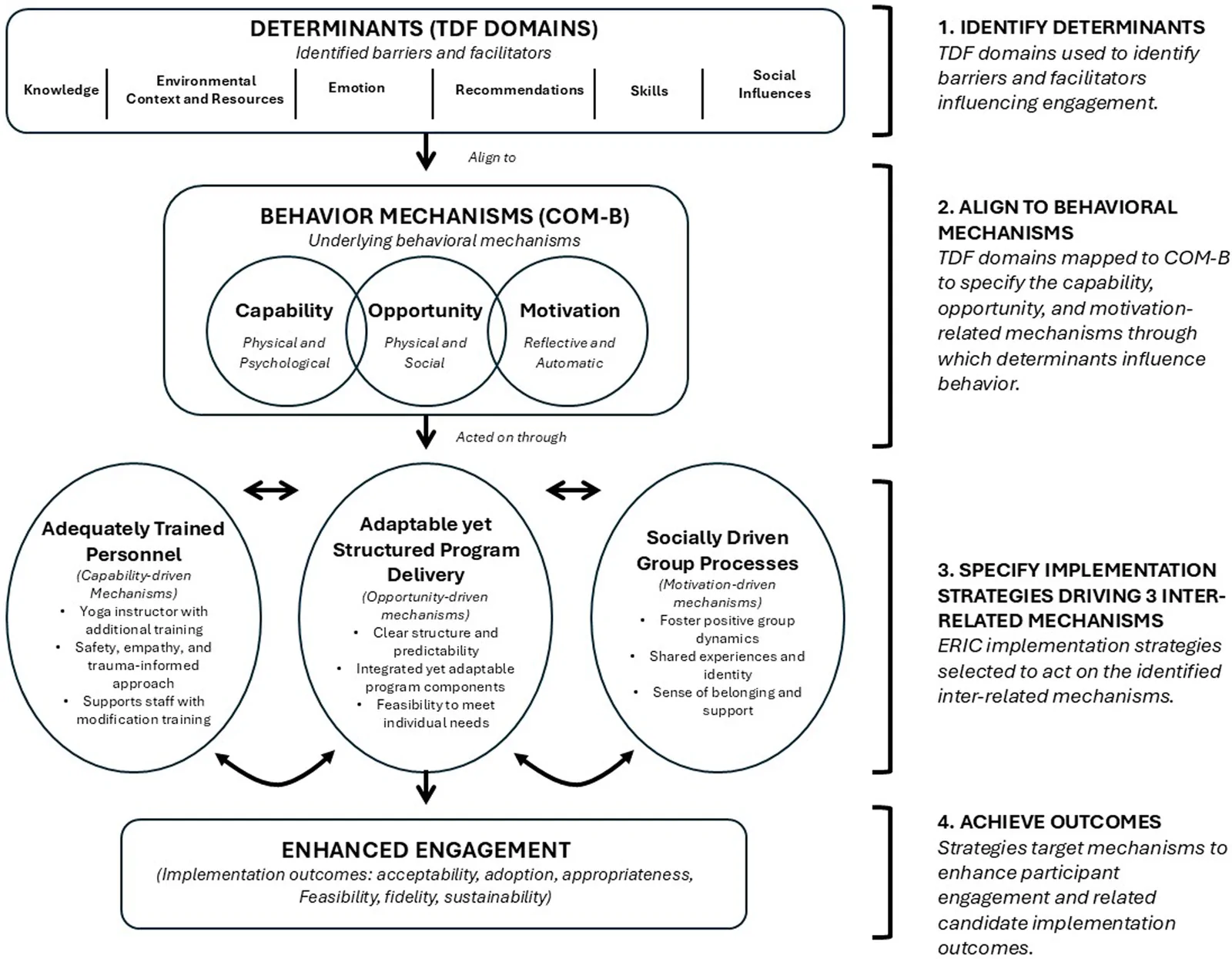
Analytic framework linking determinants (TDF), mechanisms (COM-B), and implementation strategies (ERIC) to enhance engagement and adherence to adaptive yoga for individuals with acquired brain injury.

We used interpretive analysis to move beyond TDF domain-level categorization and examined how identified determinants, aligned to COM-B components, and informed the mechanisms through which ERIC implementation strategies were expected to influence behavior. Determinants did not act independently. We identified three inter-related mechanisms, that influenced participant engagement in adaptive yoga, reflecting capability, opportunity, and motivation related processes through which implementation strategies were expected to act. These three inter-related mechanisms were: (1) adequately trained personnel, (2) an adaptable yet structured program delivery, and (3) socially driven group processes (Figure 1 and Table 3). These mechanisms reflect capability, opportunity, and motivation-related determinants that collectively positively shaped engagement in adaptive yoga.

**Table 3:** Specifying and reporting identified implementation strategies.

Participant quote	TDF domain	ERIC strategy	Specified implementation strategy	Mechanism of action	Proposed outcome	Proctor's implementation outcome
*Mechanism 1: The need for adequately trained personnel to provide physical and cognitive support*						
Denise: “I thought, ‘Oh. I can do it. No problem at all.’”	Optimism	Conduct ongoing training	Actor: Yoga instructor/team Action: Reinforce positive expectations Target: Outlook Temporality: Ongoing Dose: Repeated Outcome affected: Engagement Justification: Builds hope	Positive expectations increase engagement.	Increased participation	Acceptability
						Adherence
Denise: “I felt really safe and I could be a fragile person… that had so big impact on my emotions.”	Emotion	Promote adaptability	Actor: Yoga instructor Action: Trauma-informed delivery Target: Emotional safety Temporality: Continuous Dose: Every session Outcome affected: Retention Justification: Reduces anxiety	Safety reduces threat and supports engagement.	Increased retention	Acceptability
		Provide ongoing consultation				
Anna: “Yeah… honoring my body… we started out and move through it, it built… I really pushed myself… and it was really good.”	Skills	Use train-the-trainer strategies	Actor: Yoga instructor Action: Train yoga instructors in graded adaptive delivery Target: Instructor behavior Temporality: Pre-implementation Dose: Initial + booster Outcome affected: Fidelity Justification: Ensures proper progression	Graded delivery improves skill match and reduces participants' anxiety.	Improved participation	Fidelity
Kevin: “I liked the positive attitude that's throughout the session. I've been pleased with that”	Skills	Use train-the-trainer strategies	Actor: Yoga instructor Action: Provide coaching and encouragement Target: Participant performance Temporality: Each session Dose: Continuous Outcome affected: Engagement Justification: Builds competence	Encouragement increases confidence and participation.	Increased engagement	Fidelity
Denise: “I was very surprised that I… I said, ‘Oh, you can do this?’… I could allow myself not to be perfect.”	Beliefs about Capabilities	Provide ongoing consultation	Actor: Yoga instructor Action: Prompt reflection on success Target: Self-efficacy Temporality: During sessions Dose: Repeated Outcome affected: Engagement Justification: Reframes ability	Reframing increases willingness to engage & self-efficacy.	Increased sense of self-efficacy	Participant outcome
		Promote Adaptability				
Denise: “Yeah. You couldn't fail. You could just improve.”	Beliefs about Consequences	Promote Adaptability	Actor: Yoga instructor Action: Emphasize non-judgmental improvement Target: Outcome expectations Temporality: Each session Dose: Repeated messaging Outcome affected: Engagement Justification: Reduces fear of failure	Reduced fear increases participation & self-efficacy.	Increased sense of self-efficacy	Participant outcome
*Mechanism 2: The need for an adaptable yet structured program delivery*						
Miranda: “but I liked about it is the… consistency like you having it on specific days at specific times. Yep. I liked that. Okay, that helped me. Like keep a routine. It was super helpful.”	Environmental Context & Resources	Change record systems	Actor: Program coordinator Action: Assign single contact & standardized reminders Target: Patient communication Temporality: Enrollment → program end Dose: Each session reminder Outcome affected: Attendance/adherence Justification: Supports routine formation	Consistent cues reduce cognitive load and support routine formation.	Improved attendance	Adoption
						Adherence
Anna: “There was some familiarity and consistency with the people that were here and how it was structured.”	Environmental Context & Resources	Promote adaptability	Actor: Intervention team Action: Maintain consistent staff/structure Target: Delivery environment Temporality: Every session Dose: Continuous Outcome affected: Retention Justification: Enhances predictability	Predictability reduces uncertainty.	Increased retention	Acceptability
						Adherence
Kevin: “I had the same problem. My wife and I… GPS… we ended up where they were storing horses and goats… realizing that we were in the backside of it, and not the front.”	Environmental Context & Resources	Change physical structure and equipment	Actor: Study staff Action: Provide navigation instructions/signage Target: Access Temporality: Pre-session Dose: One-time + reminder Outcome affected: Attendance Justification: Removes barriers	Reduced navigation burden increases attendance.	Improved attendance	Feasibility
		Change service sites				
Denise: “And if I have to make a commitment for 10 weeks-it's easy to sign up for the first 8”	Knowledge	Promote adaptability	Actor: Intervention team Action: Explain intervention rolling scheduling options Target: Participant expectations Temporality: Onboarding Dose: One session + materials Outcome affected: Enrollment Justification: Aligns expectations with capacity	Clarifying expectations reduces uncertainty and increases feasibility.	Increased enrollment	Reach
		Conduct educational meetings				Adoption
Jim: “I think it would be a good system if you would say we have groups from six weeks and then there's two weeks off, and then there's another group.”	Knowledge	Promote Adaptability	Actor: Intervention team Action: Provide structured program format Target: Understanding of program design Temporality: Recruitment Dose: One explanation Outcome affected: Enrollment/retention Justification: Improves clarity	Improved understanding increases participation.	Increased enrollment and retention	Acceptability
*Mechanism 3: The need for socially driven group processes to foster engagement and adherence*						
Patricia: “I prefer to be in a group… there were like, eight of us… and that's perfect.”	Social Influence	Conduct ongoing training	Actor: Study team Action: Maintain optimal group size Target: Social dynamics Temporality: Program design Dose: Fixed groups & specific social connection integration Outcome affected: Engagement/retention & peer connection Justification: Supports belonging	Optimal group size enhances connection and participation.	Increased retention & connectedness with other participants	Acceptability
						Participant satisfaction
Kevin: “I'm going to add some yoga routines… and work with my wife… and we'll work out some things.”	Intentions	Intervene with consumers	Actor: Yoga instructor Action: Encourage carryover of skills Target: Behavior outside sessions Temporality: End of program Dose: Reinforcement discussions Outcome affected: Behavior change Justification: Promotes continuation	Planning increases likelihood of sustained behavior.	Sustained activity	Sustainability as noted by maintained patient outcomes related to balance
Barbara: “The energy from participants really makes a difference. Everyone in there is brain injured, and we’re all in there to get some type of help with that.”	Social Influences	Intervene with patients/consumers	Actor: Yoga instructor/study team Action: Foster shared identity and peer interaction Target: Group dynamics Temporality: Each session Dose: Continuous facilitation of interaction Outcome affected: Engagement/adherence Justification: Builds shared experience	Shared identity and peer interaction increase motivation and engagement.	Increased engagement and attendance	Acceptability
						Adherence
						Patient Outcome: Sense of Belonging
Anna: “There was no judgment, no guilt, no disqualification… no critical comments or anything… I really appreciated that.”	Social Influences	Provide ongoing consultation	Actor: Yoga instructor Action: Facilitate a non-judgmental group environment Target: Psychological safety within group Temporality: Each session Dose: Continuous reinforcement Outcome affected: Engagement/retention Justification: Supports emotional safety in group setting	Psychological safety reduces fear and increases willingness to participate.	Increased retention and participation	Acceptability
						Patient satisfaction
						Participant Outcome: Sense of Belonging
Anna: “The challenge with having a brain injury is finding accessible, affordable resources.”	Social Influences	Conduct local needs assessment	Actor: Study team Action: Identify need for group-based, accessible programming Target: Community context Temporality: Pre-implementation Dose: One-time assessment with ongoing refinement Outcome affected: Reach/engagement Justification: Aligns program with community needs	Addressing community need increases relevance and participation.	Increased enrollment and engagement	Reach
						Adoption

Strategies addressing the need for, and the mechanism of adequately trained personnel emphasized the role of the instructor in shaping participant experience through trauma-informed delivery, graded progression, and ongoing coaching. These strategies functioned as mechanisms of adaptive support, enabling the yoga instructor to dynamically respond to participants' physical, cognitive, and emotional needs. Participants described feelings of safety, confidence, and capability, suggesting that the adaptive yoga instructor's and assistants' behaviors directly influenced participant engagement throughout the study (Table 3).

Strategies related to the mechanism of adaptable, yet structured program delivery highlighted the importance of balancing structure with flexibility, including a standardized yoga program with modifications to meet individual abilities, consistent scheduling, clear communication, navigation supports, and flexible enrollment options. These implementation strategy elements may operate in the future as mechanisms of cognitive scaffolding, reducing confusion and cognitive burden while increasing predictability. Participants described their improved ability to plan, attend, and engage in adaptive yoga sessions because the expectations and routine were the same each week. However, when participants completed the pre- and post-assessments, they noted that the expectations were unclear and it was challenging to navigate to new locations (Table 3).

Finally, strategies targeting the mechanism of socially driven group processes underscored the value and role of in-person group-based mechanisms, including shared identity, psychological safety, and peer connection. Participants emphasized the importance of being part of a group with others who shared similar experiences, describing increased motivation and comfort in a non-judgmental environment. Our findings suggest that social processes functioned as mechanisms of action that fostered engagement, reinforcing participation and adherence through a sense of belonging and collective experience. Thus, while these social intervention elements were identified facilitators, they were not intentionally embedded or captured in the original RCT's participant outcomes. Thus, this was an important mechanism to capture because it suggests an additional participant-level outcome and highlighted a necessary intervention component, sense of belonging, to refine for a future study (Figure 1 and Table 3).

Collectively, our findings suggest that successful implementation may not be driven by any single strategy, but rather by inter-related mechanisms related to adequately trained personnel, adaptable yet structured program delivery, and socially driven group processes that together shape engagement and sustained adherence (Figure 1).

## Discussion

This study used implementation mapping to identify determinants of engagement in adaptive yoga for individuals with ABI and translate those findings into theory-informed implementation strategies. Our findings suggest that engagement is shaped by three inter-related mechanisms: (1) adequately trained personnel, (2) adaptable yet structured program delivery, and (3) socially driven group processes. Linking participant-reported determinants to TDF domains, COM-B mechanisms, and ERIC implementation strategies provides an actionable framework to inform future hybrid effectiveness-implementation trials (39).

The first mechanism of adequately trained personnel highlights the importance of hiring a yoga teacher or therapist with advanced training in delivery of yoga for people with neurological disabilities. It also underscores the importance of training each intervention assistant to address and anticipate the participant's needs by providing posture modification, physical support, and environmental setup. Across multiple TDF domains, including Skills, Emotion, and Beliefs about Capabilities, participants emphasized the importance of personnel who could provide real-time modifications, ensure physical safety, and foster psychological safety (40, 41). Implementation strategies such as train-the-trainer approaches and ongoing consultation operationalize this need by equipping personnel with the skills required to tailor intervention delivery dynamically. These findings are consistent with self-efficacy theory, suggesting that participants engaged more readily when they felt supported, safe, and capable within the intervention context (42, 43).

The second mechanism, adaptable yet structured program delivery, highlights the importance of balancing predictability with flexibility. Participants described challenges related to communication, scheduling, and navigation, which map to the TDF domains of Knowledge and Environmental Context and Resources (22). Implementation strategies such as reminder systems, clear communication materials, and flexible enrollment structures were identified to address these challenges (19, 44). These strategies may function by reducing cognitive load, increasing predictability, and aligning participant expectations with program demands. Our finding aligns with best practices for adaptive yoga for people with brain injury include “the facilitation of consistent instruction with a manual that allows for flexibility” (45). From a theoretical perspective, our findings suggest that structured delivery provides a form of cognitive scaffolding that is particularly important for individuals with ABI (44). A future hybrid design should evaluate the dose of the adaptive yoga intervention, as multiple participants reported that the initial 8-week duration felt manageable and supported enrollment, but also expressed a desire for opportunities to continue participating beyond the initial commitment.

The third mechanism of socially driven group processes appeared to foster engagement through psychological safety, shared identity, and a sense of belonging. Participants consistently emphasized the value of practicing adaptive yoga alongside others living with ABI, suggesting that social connection functioned as an implementation mechanism that reinforced motivation and sustained participation rather than simply representing a participant outcome. These findings are consistent with previous qualitative studies of adaptive yoga after ABI (8, 46–48) but extend the literature by identifying social connection as a mechanism that can be intentionally supported through implementation strategies, including peer interaction, optimal group size, and non-judgmental group environments (49, 50).

As illustrated in Figure 1, socially driven group processes interact dynamically with trained personnel and an adaptable yet structured delivery, reinforcing engagement through both relational and contextual pathways. Our findings are consistent with theories of social identity and belonging (50), suggesting that engagement in interventions is shaped not only by individual capability, but also by the extent to which participants feel connected to others with shared experiences (51–53). These findings suggest that implementation strategies should be designed and evaluated as integrated components rather than isolated actions. By operationalizing strategies according to actor, action, target, temporality, and dose, we provide sufficient detail to support replication and future hybrid effectiveness-implementation trials (20). Linking implementation strategies to candidate implementation outcomes (e.g., acceptability, adoption, appropriateness, feasibility, fidelity, sustainability) (54) further advances current recommendations for specifying implementation mechanisms and contextual fit (20, 25).

### Limitations

This study was conducted as part of a pilot RCT with a small, self-selected sample recruited from a single geographic region, which may limit the transferability of findings. Because only intervention completers were interviewed, determinants influencing early withdrawal or poor attendance were not fully captured. Similarly, the absence of perspectives from the yoga interventionist, intervention assistants, study personnel, care partners, and participants who discontinued the intervention may have limited the comprehensiveness of the identified implementation strategies. Although the proposed implementation strategies and mechanisms of engagement may have broader applicability, they remain untested beyond this study. Finally, the primary deductive analysis guided by the TDF may have constrained the identification of determinants beyond the framework.

### Implications for implementation science and future research

These findings inform the design of future hybrid effectiveness-implementation trials evaluating adaptive yoga for individuals with ABI. Future studies should test whether the identified implementation strategies improve participant engagement and implementation outcomes while incorporating perspectives from interventionists, care partners, study personnel, and participants who discontinue interventions. Evaluating these mechanisms across different rehabilitation settings and stages of recovery will further refine implementation strategies and improve scalability.

## Conclusions

Findings from this study demonstrate that engagement in adaptive yoga for individuals with acquired brain injury is shaped by dynamic, inter-related mechanisms rather than discrete determinants. By linking participant-reported experiences to TDF domains, COM-B mechanisms, and ERIC implementation strategies, we provide a theory-informed approach to translating qualitative data into actionable strategies. The three identified mechanisms: (1) trained personnel, (2) adaptable yet structured delivery, and (3) socially driven group processes, highlight critical components for optimizing participant engagement and sustained participation. These insights inform the design of a future hybrid trial and underscore the importance of explicitly specifying mechanisms of action to enhance implementation success in adaptive yoga interventions.

## Data Availability

The raw data supporting the conclusions of this article will be made available by the authors, without undue reservation.
